# User Requirements and Perceptions of a Sensor System for Early Stress Detection in People With Dementia and People With Intellectual Disability: Qualitative Study

**DOI:** 10.2196/52248

**Published:** 2024-06-21

**Authors:** Esmee Adam, Franka Meiland, Noud Frielink, Erwin Meinders, Reon Smits, Petri Embregts, Hanneke Smaling

**Affiliations:** 1 Department of Public Health and Primary Care Leiden University Medical Center Leiden Netherlands; 2 University Network for Care sector Zuid-Holland Leiden University Medical Center Leiden Netherlands; 3 Department of Medicine for Older people Amsterdam University Medical Centers Amsterdam Netherlands; 4 Tranzo Tilburg School of Social and Behavioral Sciences Tilburg University Tilburg Netherlands; 5 Mentech Innovation B.V. Eindhoven Netherlands

**Keywords:** stress detection, sensor system, garment integrated, wearable, user requirements, dementia, intellectual disability, intellectual disabilities, long-term care, perceptions, wearables, qualitative study, residents, communication impairment, impairments, garment sensor

## Abstract

**Background:**

Timely detection of stress in people with dementia and people with an intellectual disability (ID) may reduce the occurrence of challenging behavior. However, detecting stress is often challenging as many long-term care (LTC) residents with dementia and residents with ID have communication impairments, limiting their ability to express themselves. Wearables can help detect stress but are not always accepted by users and are uncomfortable to wear for longer periods. Integrating sensors into clothing may be a more acceptable approach for users in LTC. To develop a sensor system for early stress detection that is accepted by LTC residents with dementia and residents with ID, understanding their perceptions and requirements is essential.

**Objective:**

This study aimed to (1) identify user requirements for a garment-integrated sensor system (wearable) for early stress detection in people with dementia and people with ID, (2) explore the perceptions of the users toward the sensor system, and (3) investigate the implementation requirements in LTC settings.

**Methods:**

A qualitative design with 18 focus groups and 29 interviews was used. Focus groups and interviews were conducted per setting (dementia, ID) and target group (people with dementia, people with ID, family caregivers, health care professionals). The focus groups were conducted at 3 time points within a 6-month period, where each new focus group built on the findings of previous rounds. The data from each round were used to (further) develop the sensor system. A thematic analysis with an inductive approach was used to analyze the data.

**Results:**

The study included 44 participants who expressed a positive attitude toward the idea of a garment-integrated sensor system but also identified some potential concerns. In addition to early stress detection, participants recognized other potential purposes or benefits of the sensor system, such as identifying triggers for challenging behavior, evaluating intervention effects, and diagnostic purposes. Participants emphasized the importance of meeting specific system requirements, such as washability and safety, and user requirements, such as customizability and usability, to increase user acceptance. Moreover, some participants were concerned the sensor system could contribute to the replacement of human contact by technology. Important factors for implementation included the cost of the sensor system, added value to resident and health care professionals, and education for all users.

**Conclusions:**

The idea of a garment-integrated sensor system for early stress detection in LTC for people with dementia and people with ID is perceived as positive and promising by stakeholders. To increase acceptability and implementation success, it is important to develop an easy-to-use, customizable wearable that has a clear and demonstrable added value for health care professionals and LTC residents. The next step involves pilot-testing the developed wearable with LTC residents with dementia and residents with ID in clinical practice.

## Introduction

Challenging behavior is prevalent in long-term care (LTC) residents, including people with dementia and people with an intellectual disability (ID) [1,2]. Challenging behavior can be defined as “behavior of such an intensity, frequency or duration as to threaten the quality of life and/or the physical safety of the individual or others and is likely to lead to responses that are restrictive, aversive or result in exclusion” [3]. Examples of challenging behavior include aggression, apathy, depression, and resistance to care. Approximately 10%-25% of people with ID and 80%-90% of nursing home residents with dementia display a form of challenging behavior [1,2]. Challenging behavior has negative consequences for the quality of life of the person expressing the challenging behavior, due to an increased risk of physical injury, the use of restrictive practices, and social isolation [4,5]; has negative consequences for the well-being of other residents [2,6,7]; and is burdensome for caregivers [8-10]. Furthermore, challenging behavior is persistent and increases in severity over time [11-13].

The cause of challenging behavior is complex and involves an interplay of factors, including reduced stress-coping mechanisms and increased vulnerability to stress in people with dementia and people with ID [14,15]. Failure to recognize and address stress buildup in a timely manner can lead to challenging behavior and its associated negative consequences. Early and effective detection, intervention, and prevention of stress have the potential to reduce the occurrence of challenging behavior [16], which in turn may alleviate the burden on caregivers and improve residents’ quality of life. However, timely detection of stress is often challenging due to communication impairments commonly observed in LTC residents with dementia and residents with ID, limiting their ability to express themselves in ways that are recognizable for their caregivers.

Technology can play a vital role in assisting caregivers to detect stress in people who have difficulties expressing themselves. By using physiological parameters, such as heart rate, skin conductance, temperature, and respiration [17-20], technological aids can effectively identify early signs of stress in people with dementia [21,22] and people with ID [23,24]. These aids typically take the form of small electronic wearable devices that continuously monitor the users’ physiological parameters. The use of wearables for early stress detection enables caregivers to promptly identify stress-inducing factors and respond to the needs of the person with dementia or ID more effectively [22].

Currently, a broad range of wearables for stress detection is available [25]. However, user acceptance of existing wearables for early stress detection is not always favorable. That is, several wearables are reported to be uncomfortable or difficult to use [26-28], which can be particularly challenging for individuals with sensory processing difficulties and potentially increase their stress levels. Integrating sensors into clothing items may offer a more comfortable and acceptable solution compared to traditional wearables [26]. Moreover, the integration of sensors into textiles enables accurate measurement of stress levels [29], and successful application to mitigate stress in people with ID has been shown [30].

The success of implementing technologies in LTC is influenced by various factors, including the acceptability of the technology to residents, the behavior of family caregivers, and the willingness of staff to adopt the technology [31,32]. It is worth noting that a significant proportion of innovations, estimated to be between 30% and 90%, fail during implementation [33]. The likelihood of successful implementation increases when a technology meets the requirements of its specific users [31]. Therefore, involving stakeholders early in the technology development process is crucial.

To be able to develop a wearable for a sensor system for early stress detection in people with dementia and people with ID that is accepted by and meets the specific needs of its users, it is important to explore their perceptions and user requirements. This study aimed to (1) identify user requirements for a garment-integrated sensor system for early detection of stress in people with dementia and people with ID, (2) explore the perceptions of users (ie, persons with dementia, persons with ID, family caregivers, and health care professionals) toward the sensor system, and (3) investigate the requirements for implementation in LTC. We used the requirements collected in this study to develop the wearable and make it compatible with the HUME stress detection system (Mentech Innovation). HUME uses trained artificial intelligence models to convert real-time physiology signals (e., heart rate, electrodermal activity) collected with sensors in wearables into stress predictions [29,34,35]. The balanced accuracy of stress detection with these wearables is 80% [29]. The stress prediction can be displayed on a smart phone or in a dashboard analysis tool [36].

## Methods

### Study Design

This study used a qualitative design with online focus groups and interviews. Focus groups were chosen as the main method as they offer participants an opportunity to share their ideas and to interact and complement each other [37]. Interviews were conducted with community-dwelling people with dementia and 1 family caregiver with hearing difficulties, as online focus groups were not feasible for them. Most persons with dementia did not feel confident enough to join an online focus group.

**Figure 1:**
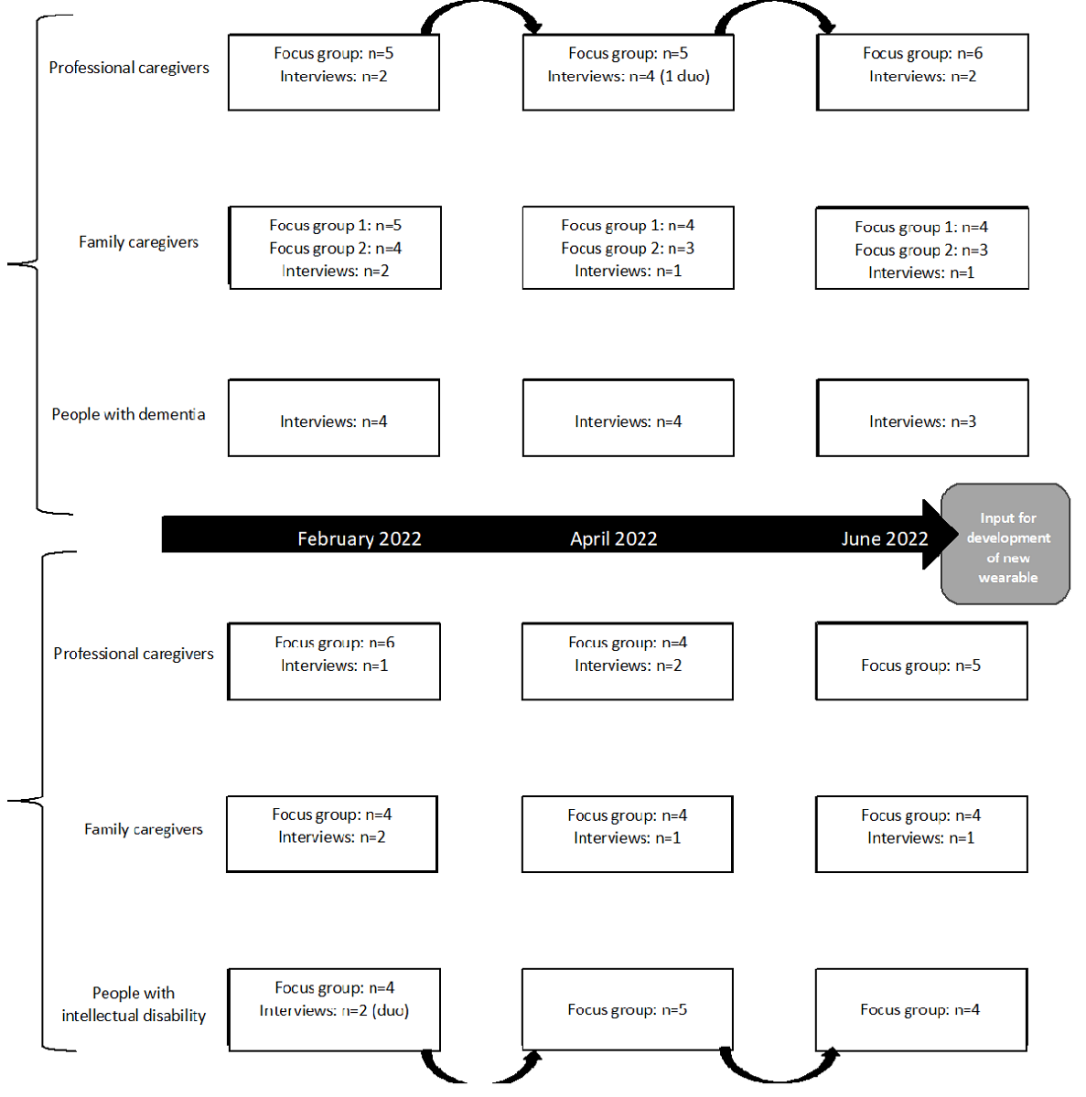
Overview of participants per data collection round per setting.

The focus groups were organized per setting (dementia and ID) and per group: (1) people with dementia or mild ID, (2) health care professionals, and (3) family caregivers (see Figure 1). The focus groups were conducted online through Microsoft Teams at 3 time points within a 6-month period. This allowed for in-depth discussions per topic and for each new focus group to build on the findings of previous rounds. The latter was especially important because the development process of the sensor system ran parallel to the focus groups. Data from each round were used to (further) develop the sensor system. The focus groups provided direct interaction with the end users. We used these moments to iterate on design and functionality. At the same time, design choices, user requirements, and systems requirements were confirmed and adjusted, if needed. In addition, the barriers to implementation and scaling were revisited. Participants could provide feedback on sensor system prototypes and types of garments as the development process progressed. For example, 3 types of textiles for the garment were developed. In the next round, the participants were asked to rank the textiles and explain their preference. This led to the choice of the fabric for the garment. A description of the technological development of the sensor system was not within the scope of this study and therefore not included. Online focus groups were chosen due to COVID-19 restrictions in the Netherlands at the start of the first focus group and because of previous positive experiences with online focus groups [38]. The interviews were conducted at the interviewee’s location of preference, which was usually online or at their home (persons with dementia). Data were collected between February and July 2022. All participants received a gift card as a token of appreciation.

### Ethical Considerations

This study was conducted according to the principles of the Declaration of Helsinki [39]. The Leiden-The Hague-Delft Medical Ethical Committee reviewed the study protocol and provided a waiver of medical ethical approval (N21.148) since the study was not subject to the Dutch Medical Research Involving Human Subjects Act (WMO). Participation was confirmed after participants signed the informed consent form.

### Study Sample and Recruitment

Participants were asked to participate in all 3 focus groups. Both participants with dementia and family caregivers of people with dementia were recruited via the Dutch Alzheimer society (Alzheimer Netherlands). Alzheimer Netherlands sent an email about the study to its panel members. Interested members could leave their email address and phone number for the researcher to contact them. An information package was sent via email. After 2 weeks, the researcher phoned the people with dementia to explain the study and to discuss their preferred form of participation (focus group or interview). Family caregivers were contacted by a researcher via email, with a follow-up call, if needed. Two people (a health care professional and a family caregiver) dropped out due to unknown reasons after signing the informed consent form.

Health care professionals from the dementia setting were recruited from 5 health care organizations via the University Network for the Care sector Zuid-Holland (UNC-ZH). The scientific coordinator of the UNC-ZH sent an information package consisting of a flyer, information letter, and informed consent form via email to the collaborating health care organizations, asking health care professionals to participate. They also placed a call on the UNC-ZH social media. Finally, the research team recruited through its personal (professional) network.

All participants from the ID setting were recruited from 6 health care organizations via the Academic Collaborative Center Living with an intellectual disability (AWVB, Tranzo, Tilburg University) or via the 2 health care organizations that were part of the research collaboration. The contact person of the AWVB sent an information package consisting of a flyer, information letter, and informed consent form via email to the collaborating health care organizations, asking health care professionals, family caregivers, and people with mild ID to participate. If a person wanted to participate, they were asked to sign the informed consent form and send it back to the researcher.

Criteria for inclusion were (1) age≥18 years, (2) a sufficient understanding of the Dutch language, and (3) ability to give consent to participate in the study. Additionally, health care professionals had to have worked with people with dementia or people with ID for at least 6 months. Family caregivers had to provide care for a relative with dementia or ID for at least 2 hours per month. Community-dwelling people with dementia (diagnosed by a physician) had to have a sufficient language level to be able to communicate and had been informed about and had to be aware of their diagnosis, while people with ID had to have mild ID (intelligence quotient [IQ] 50-70).

### Procedure

A detailed overview of focus groups and interviews per subgroup is depicted in Figure 1. Focus groups were conducted with 4-6 participants per group to optimize online interactions; 2 focus groups had 3 participants due to last-minute cancellations. To ensure the inclusion of all participants’ perceptions in the study, those who could not attend the focus group were offered an opportunity to participate in an online interview later. One person with dementia was not able to participate in the last interview round due to personal circumstances. Prior to the first focus group or interview, participants received a link to a brief online questionnaire in Castor’s electronic data capture (EDC) system [40] to check whether they met the inclusion criteria and to collect participant characteristics for the sample description.

The focus groups and interviews were semistructured, resulting in a higher level of standardization, while allowing the researchers to ask follow-up questions, when necessary [41]. An initial interview was conducted with 2 people with mild ID to pilot the questions for the focus groups. Minor adaptations were made to the guides, depending on the subgroup (eg, topics, such as data security and implementation, were discussed more briefly and with less follow-up questions for people with ID and people with dementia).

Each focus group started with an introduction explaining the goal and procedure of the meeting. An overview of topics per focus group round is depicted in Textbox 1. Rounds 2 and 3 started with the moderator presenting the main results of the previous round and inviting the participants to respond and ask questions. In this way, participants had an opportunity to indicate whether the results (still) reflected their opinions and to supplement the results, if needed. Topics were divided per focus group in an organic and chronological order and from broad topics to more specific topics, starting with the perceptions of participants and ending with conditions for implementation.

All focus groups were moderated by the first or the last author and an assistant moderator (research assistant; medical and psychology students). The moderator introduced the topics in each focus group and encouraged participants to share their thoughts and stimulate discussion. The assistant moderator was responsible for time management and provided technical assistance. Each focus group lasted between 90 and 120 minutes. The assistant moderator made field notes that included observations of the process, reflections of the moderator and assistant, and the main results.

Interviews were conducted by the first author or a research assistant (medical or psychology students) using an interview guide composed by the first and the last author. Interviews lasted between 30 and 55 minutes. Interviews with people with dementia lasted between 50 and 75 minutes, as the interview pace was calmer, with room for repetition of questions, when needed.

**Overview of topics per focus group or interview round.**

**Round 1**

- Introduction and explanation of the study
- Personal experiences with stress and challenging behavior
- Perceptions, (dis)advantages, and concerns regarding the sensor system
- Requirements for use
- Requirements and preferences concerning the garment (eg, what kind of garment, location of sensors, alternative ideas)

**Round 2**

- Introduction and study aims
- Presentation of main results of round 1
- Choosing a garment from the most popular options from round 1
- Requirements and preferences regarding the fabric and alarm signal
- Data security and privacy

**Round 3**

- Introduction and study aims
- Presentation of main results of round 2
- Refining the sensor system and alarm (eg, preferred battery life, preferred visualization of the alarm)
- Ethical considerations (eg, stigmatization, autonomy)
- Implementation in long-term care (LTC)

### Data Analysis

The focus groups were audio-recorded and transcribed verbatim. A thematic analysis with an inductive approach was used to analyze the data [42]. The first and the last author familiarized themselves with the data by reading and rereading the transcripts. The researchers then identified relevant text segments (eg, text segments that included data regarding the development of the sensor system, perceptions about the sensor system, and implementation of the sensor system in LTC) and then independently coded those segments using the first 2 focus group transcripts (1 ID and 1 dementia setting). The researchers then compared their codes and reached consensus, resulting in an initial codebook. The design of the codebook and the analysis approach were discussed in the project team. The consensus codebook was used to recode the first 2 focus group transcripts. The remaining transcripts were independently coded by 2 researchers (the first author and 1 of 3 research assistants with a medical background). During the coding, new codes were added to the codebook, when necessary. The researchers compared their coding of every transcript and reached consensus together. If consensus was not reached between the 2 researchers, a third researcher (the last author) was consulted. Each transcript from the interviews was considered as additional data and was analyzed alongside the focus group data. After all the transcripts were coded, the first author searched for themes, which were subsequently reviewed and named with the help of the last author. The final themes and reporting of results were discussed with the research team. It should be noted that we did not find notable differences in data richness or thematic emergence between the 2 data sources. All transcripts were coded using the software program ATLAS.ti version 22 [43].

## Results

### Participant Characteristics

A total of 18 focus groups and 29 interviews were conducted. Of the 44 participants, 23 (52.3%) were from the dementia setting and 21 (47.7%) from the ID setting. Health care professionals from both settings were physicians (n=4, 23.5%), nurses (n=3, 17.6%), psychologists (n=1, 5.9%), behavioral experts (n=2, 11.8%), (case or team) managers (n=3, 17.6%), and support staff (n=4, 23.5%). Health care professionals had experience with different levels of ID and different forms of dementia. Family caregivers took care of their partner (n=5, 50%) or parent(s) (n=5, 50%) with dementia or of a son/daughter (n=4, 66.7%), cousin (n=1, 16.7%), or sibling (n=1, 16.7%) with ID. Table 1 presents the participant characteristics per setting.

**Table 1:** Participant characteristics.

Characteristics			Dementia setting							ID^a^ setting				
		People with dementia (n=4)		Family caregivers (n=10)		Health care professionals (n=9)		People with ID (n=7)			Family caregivers (n=6)		Health care professionals (n=8)	
Age (years), mean (SD, range)			66.3 (9.8, 55-75)		64.3 (7.1, 54-72)		49.0 (13.4, 27-65)		40.7 (15.6, 25-62)			55.7 (12.2, 39-73)		36.6 (11.1, 22-52)
Female, n (%)			2 (50.0)		7 (70.0)		9 (100.0)		2 (28.6)			4 (66.7)		8 (100.0)
**Education level^b^, n (%)**														
	Low	2 (50.0)		—^c^		—		5 (71.4)			—		—	
	Medium	—		5 (50.0)		1 (11.0)		—			—		1 (12.5)	
	High	2 (50.0)		5 (50.0)		8 (89.0)		—			6 (100.0)		7 (87.5)	
	Unknown	—		—		—		2 (28.6)			—		—	
Work experience with target group (years), mean (SD, range)			—		—		16.6 (15.1, 3-45)		—			—		9.4 (8.6, 2-26)
**Type of dementia of participant or their relative, n (%)**														
	Alzheimer’s disease	2 (50.0)		3 (30.0)		—		—			—		—	
	Vascular dementia	1 (25.0)		2 (20.0)		—		—			—		—	
	Mixed type^d^	—		3 (30.0)		—		—			—		—	
	Unknown	1 (25.0)		—		—		—			—		—	
	Frontotemporal dementia	—		2 (20.0)		—		—			—		—	
**Severity of ID of participant or their relative, n (%)**														
	Profound	—		—		—		—			2 (33.4)		—	
	Mild	—		—		—		7 (100.0)			—		—	
	Moderate	—		—		—		—			1 (16.7)		—	
	Severe	—		—		—		—			2 (33.4)		—	
	Unknown	—		—		—		—			1 (16.7)		—	
Technology experience (yes), n (%)			2 (50.0)		7 (70.0)		7 (78.0)		1 (14.3)			4 (66.7)		6 (75.0)
Technology experience (years), mean (SD, range)			16 (—, 12-20)		6.9 (10.3, 1-30)		9 (8.4, 2-22)		23 (—,—)			8.3 (8.2, 2-15)		6.5 (5.9, 2-15)

^a^ID: intellectual disability.

^b^Low: (special) primary education, prevocational education, vocational education level 1, senior general secondary education grades 1-3, preuniversity education grades 1-3, trade school; medium: senior general secondary education grades 4-5, preuniversity education grades 4-6, vocational education levels 2-4; high: university of applied sciences, university.

^c^Not applicable.

^d^Mix of Alzheimer’s disease and vascular dementia or Lewy bodies, including Parkinson’s disease.

### User Requirements for the Sensor System

The user requirements for the sensor system were divided into 2 themes: (1) requirements for user acceptance (form, fabric, customizability, visibility, and user friendliness) and (2) basic system requirements.

#### Requirements for User Acceptance

##### Form

Most participants were positive about the idea of integrating the sensor system into clothing. A T-shirt or top was suggested by participants from all groups. Socks, wristbands, and undergarments were also suggested by participants in most focus groups. The main reason for choosing these clothing items was familiarity: most people wear these items every day, so it would not be a completely new experience and will, therefore, increase user acceptance.

A sensor that he has now, a chest strap, like a heart rate monitor. Well, it's quite a hassle to put it on. And if he already doesn't feel good in the morning, then it's just impossible to get the thing on. While your socks and your shoes and your clothes—that's all in his everyday routine, so he recognizes that. And it will be a little bit easier for him to accept that than something less familiar to him.

Family caregiver 6, ID setting

A bra and undergarments were only mentioned by family and professional caregivers from both settings. Furthermore, the idea of integrating a sensor system into a Band-Aid instead of clothing was suggested by participants from 4 different focus groups (health care professionals from both settings and both groups of family caregivers from the dementia setting).

##### Fabric

Participants indicated that the fabric of the sensor-integrated garment should be comfortable, breathable to prevent excessive sweating, and preferably made from natural materials to ensure user acceptance. Cotton with a certain amount of stretch was preferred, as that fabric would be familiar to most residents. Wool was regarded as less suitable due to potential irritation or allergic reactions. To accommodate individuals with ID who may be hypersensitive or have difficulties with sensory processing, participants thought it is crucial for the garment to be designed with minimal folds, seams, creases, and labels. Furthermore, the garment should stay firmly in place and should not move, providing a consistent and comfortable experience.

##### Customizability

Participants from all groups emphasized the importance of customizable clothing to increase user acceptance. They indicated that the garment should be similar to familiar clothing items and something residents wear often. In addition, participants expected that unfamiliar clothing that is not tailored to the needs and preferences of the user may cause distress. An alternative option that was suggested was to integrate the system into the residents’ own clothing. However, participants expected that this could lead to a potential decrease in reliability of the system, which made it a less favorable option. Therefore, participants believed that multiple types of garments should be available. The option to choose one’s own garment was especially important for people with mild ID.

For our clients, being in charge is incredibly important; they consider that very important. So, the mere fact that they have a choice is important to them.

Health care professional 7, ID setting

##### Visibility

When incorporating the sensor into the clothing, it is important for the sensor to be discreet and unnoticeable to the user, allowing for uninterrupted use without causing any discomfort or distraction. Moreover, people with mild ID and people with dementia mentioned that it is important that the sensor system not be visible to others.

I think it should be hidden. Because otherwise, you run the risk of being labeled, like, “look, he can't go anywhere on his own.”

Person with mild ID 7, ID setting

Participants expected visibility not to be an issue for people with advanced dementia or severe ID, as they would not be aware of potential stigmatization.

##### User Friendliness

Caregivers indicated that the sensor system itself should be intuitive and simple to use and that the garment should be easy to put on in order to avoid stress in both residents and health care professionals.

It just has to be as simple as possible. I'm thinking about those organizations. There are so many on-call workers, freelancers, and flex workers, and they also have to deal with these things. But you really have to start from the weakest link in such a team who has to be able to put it on. If the whole thing becomes very “rocket science,” then it's doomed to fail.

Health care professional 5, ID setting

#### Basic System Requirements

According to participants, the sensor system needs to meet several practical conditions. These requirements are presented in Table 2.

In all participant groups, except people with ID, participants indicated that the sensor system should preferably contain 2 batteries so that it can still be used while 1 battery is charging. Furthermore, health care professionals and family caregivers from both settings thought that the battery should last at least 24 hours to avoid an additional burden on the caregivers, to enable monitoring day and night (if needed), and to enable health care professionals to recognize patterns in stress buildup throughout the day (and night).

[...] or because you have to change a battery too often. These are all reasons why you might not use something.

Family caregiver 4, dementia setting

Participants stressed that the sensor system must be reliable, not prone to failure, and not just rely on a local Wi-Fi connection (Wi-Fi is not always stable in some LTC facilities). Furthermore, the system must be self-learning, and its stress-detecting model must be personalized to its user. Participants from both settings indicated that ideally, the system would be combined with health technologies that the resident already uses.

Because there are also people who have an incontinence sensor during the night, and if you then also burden these people with a stress sensor, that would be a bit too much. You also need to be careful you don't turn a patient into a kind of robot that you only attach cables to.

Health care professional 6, dementia setting

Depending on the situation, the receiver of the alarm signal in the case of stress buildup should be adjustable. All participant groups indicated the importance of using an ascending scale to indicate the level of stress. This would allow for easy interpretation of a resident’s stress level (or own stress levels in the case of people with dementia or with ID) and to (re)act accordingly if the stress level exceeds a certain threshold. Family and professional caregivers suggested linking the scale to the different levels in the residents’ personal treatment and stress management plan in LTC.

According to health care professionals, the storage period of the yielded data should match the storage period of the electronic patient files (20 years after the last modification). Several family caregivers suggested a storage period of 1 year to enable health care professionals to monitor the influence of seasons and holidays on the resident’s stress levels. Participants with dementia or with ID expressed mixed opinions regarding the storage of their data. Some participants expressed discomfort with the idea of storing their data for longer than a day, particularly when using the sensor system in home settings. They preferred the system to be used for short-term monitoring rather than continuous data retention. Other participants mentioned that data could be stored for longer periods, especially when residing in a nursing home. They acknowledged the potential benefits of being able to retrospectively review and detect trends over time.

**Table 2:** Practical system requirements for a garment-integrated sensor system.

Category	Requirements
System	Waterproof and washable (≥40°C) to ensure proper hygiene and user safety. Sustainable to limit costs and waste. Durable—withstand users trying to take it apart. Wireless and include a rechargeable battery.
Technology	Reliable system with good sensitivity and specificity. Self-learning system. Ideally integrate with other technologies and with electronic patient files.
Location	Sensor on feet to measure skin conductance or torso for heart rate measures. Other measurement location options, such as ankle, arm, leg, neck, and wrist.
Alarm	Whether the resident receives an alarm depends on the cognitive level of the wearer. People with severe ID^a^ or dementia should not receive or notice an alarm as this can add stress. Option to personalize alarm; visual with smileys, thermometer, graphs and colors, or auditive or vibrating alarm. Alarm must be received on the smartphone. The iPad or smartwatches are less frequently suggested alternatives.
Safety	The system must not contain loose parts in order to prevent choking hazards. The system should be out of reach of the resident to prevent them from breaking it and causing harm to themselves or others. Suitable for delicate, sensitive skin of older people. The system must not emit harmful radiation.
Data security	Clear agreements must be made about the use of data and their purpose in the care process. Data must be properly secured and only accessible to health care professionals and others (eg, schoolteachers or family members) working directly with the resident. Participants differed on whether flex workers should also have access to data. Data should be stored for a limited period ranging from a week to several years. The storage period should be discussed with and agreed upon by family caregivers (and, if possible, the client) before using the sensor system. The developer of the sensor system should not be the owner of the yielded data. The developer may receive anonymized data solely for the purpose of improving the system.

^a^ID: intellectual disability.

### Perceptions of Users Regarding a Sensor System for Stress Detection

The participants mentioned potential advantages and disadvantages of using a garment-integrated sensor system for early stress detection. The perceived advantages and disadvantages mentioned in all focus groups are presented in Textbox 2.

Health care professionals from both settings indicated that the sensor system itself would not provide information about the cause of stress in the resident. However, participants from all groups, except people with mild ID, did expect that using the sensor system for a longer period could lead to more knowledge concerning stress and challenging behavior in people with ID and people with dementia. Some health care professionals from both settings and family caregivers from the dementia setting expected that the new insight into the triggers of stress in residents could be confronting for health care professionals. Health care professionals could discover that their way of working or even their own stress level induced stress in some of the residents. This was perceived as a disadvantage by some participants as it could lead to friction within the team and as an advantage by others as it could provide an opportunity to create more calmness and increase the resident’s well-being by considering the influence of one’s own emotional state or stress level. Health care professionals and family caregivers from both settings also expected better communication between staff and family due to objective measures and more trust and calmness from family as they expect health care professionals to be able to better respond to the needs of their relative. Participants from all groups expected residents to feel more understood by family caregivers and health care professionals.

**Overview of perceived advantages and disadvantages of a garment-integrated sensor system.**

**Perceived/expected advantages**

- Identifying (triggers/causes of) stress (mentioned in all participant groups)
- Early intervention and prevention of challenging behavior (mentioned in all participant groups)
- Increase in self-regulation of residents with mild dementia or with intellectual disability (ID) due to recognition of their emotions (mentioned in all participant groups)
- Better insight into persons with communication difficulties (mentioned in all participant groups)
- Objective measure (mentioned in 5 of 6 groups)
- More insight into challenging behavior and how to best deal with this (mentioned in 5 of 6 groups)

**Perceived/expected disadvantages**

- Technical issues, such as malfunction or false alarms (mentioned in all participant groups)
- System is not accepted by the users (mentioned in all participant groups)
- Replacing the human aspect of care (mentioned in 5 of 6 groups)
- False-positive alarms caused by a medical condition or positive stress (mentioned in 5 of 6 groups)
- Could be lost in laundry or broken (mentioned in 5 of 6 groups)

Participants recognized that various groups could benefit from the stress-detecting sensor system, including individuals with dementia or ID residing in LTC facilities or living in the community. They particularly highlighted potential benefits for those experiencing rapid mood swings, nighttime restlessness, or communication impairments. Participants expected the use of the sensor system to be beneficial in more areas than just early stress detection. Health care professionals from both settings expected the sensor system to be used to evaluate (the effect) of interventions targeting challenging behavior. Moreover, health care professionals also expected that the data yielded with the sensor system would supplement electronic patient records to create a more complete overview of the resident’s status. In the dementia setting, participants expected a reduced need for medication.

You can avoid getting into the whole medication thing. And I would really like that. Because sometimes, the Haldol is turned to very quickly. And if we can prevent that, that a health care professional can get to someone in time to regulate the stress, to divert the stress, and so not end up in the medication circuit, that would be worth a lot to me.

Family caregiver 11, dementia setting

Disadvantages of the system included the fear of technology replacing human contact. This fear was expressed by all participants groups, except people with mild ID. Participants mentioned that using the sensor system could contribute to the digitalization of health care and reduce the resident to data, graphs, and alarms. Moreover, the job satisfaction of health care professionals could be affected if they only have to respond to alarms. In addition, participants expressed a fear that residents without stress could be “forgotten.”

Will health care soon be based on “to measure is to know”? That people will only rely on that and no longer even see the actual signs so that they say, “Yes, but look, she is doing very well. She is not stressed, just look at the device.” And then when you, as a family carer, say, “Look at those eyes. She is smiling, but those eyes are saying something else,” I think that would really be a shame.

Family caregiver 5, dementia setting

According to health care professionals from both settings and family caregivers from the ID setting, the role of a health care professional could change. Health care professionals are expected to use the sensor system and interpret the alarm. This requires knowledge and new skills. Some health care professionals and family caregivers from the ID setting indicated that this might feel like an extra burden for the already busy health care professionals. However, participants expected that using the sensor system may lead to a more relaxed way of working because health care professionals can better assess the resident’s emotional state. This contradiction was summarized by a participant as follows:

Couldn't it work both ways? That someone thinks “I understand better now how a person is put together in terms of their stress pattern” and therefore feels that you can contribute something at an earlier stage to regulate that stress, or the opposite: “I don't have enough time, and now half of the people here are stressed...so the stress I see on the phone just stresses me out more.”

Health care professional 8, ID setting

Health care professionals from the dementia setting expected to switch from a reactive working style to a more preventive way of working. All participants from the dementia setting and family caregivers from the ID setting expected that using the sensor system could help reduce the burden on caregivers.

### Requirements for and Barriers to Implementation

Participants shared their ideas on the requirements for successful implementation in LTC (Table 3), as well as potential barriers.

Regarding barriers to implementation, participants felt that staff shortage and the high number of temporary workers could hamper proper implementation of the sensor system. A lack of acceptance by residents and health care professionals perceiving the sensor system as a burden was also indicated as a barrier. The same may apply when not all professionals support the use of the sensor system. This could lead to friction within the team or between family and professional caregivers. However, if health care professionals do see the added value of the system, participants expected that health care professionals would be willing to go the extra mile to make it a success.

Privacy and data security were important topics for participants in all focus groups. Several participants indicated that every Dutch LTC facility should be legally obligated to comply with certain privacy requirements. Participants expected that this will protect residents who are wearing the sensor system. Most participants did not consider the detection of the heart rate or skin conductance as an invasion of privacy. Nevertheless, all participants indicated that this information must be properly secured, must be impossible to trace back to an individual, and can only be referred to by professional caregivers working directly with the resident. Overall, privacy proved to be of great importance to all participants and was perceived as a delicate subject that could be a barrier.

**Table 3:** Requirements for implementation of the sensor system in LTC^a^.

Requirement	Description
Added value	Using the sensor system must have an added value for resident and/or health care professional.
Initiator	One person must be dedicated to initiating the use of the sensor system by residents or their family caregivers. In the ID^b^ setting, this could be behavioral experts. In the dementia setting, this should be discussed in multidisciplinary meetings.
Permission for use	Using the sensor system must be a well-considered choice of the wearer or their representative. Permission must be asked from the resident or legal representative or both. In case the resident is unable to express their opinion, health care professionals and family caregivers need to look for signs of resistance in the person wearing the sensor system and respond accordingly.
Costs	The cost of the system should be reimbursed by the health insurer or the health care organization. If reimbursement is not feasible, a lease plan is an acceptable alternative.
Education	All stakeholders must be educated about the sensor system. A trial period is desirable. There should be a protocol that describes the proper use of the system.

^a^LTC: long-term care.

^b^ID: intellectual disability.

## Discussion

### Principal Findings

This study examined the user requirements for a to-be-developed garment-integrated sensor system (wearable) for early detection of stress in people with dementia and people with ID, determined the perceptions of users regarding the sensor system, and explored the requirements for implementation in LTC. Overall, participants showed a positive attitude toward the concept of a garment-integrated sensor system for early stress detection in people with dementia and people with ID. To enhance user acceptance, the sensor system must fulfill both basic system requirements (eg, be waterproof and safe) and specific user requirements (eg, customizability and user friendliness). Moreover, several requirements, such as a clear added value of the system for LTC residents, education to ensure proper use of the system, and potential barriers (eg, staff shortage), must be considered when implementing the garment-integrated sensor system in LTC.

Stakeholders were positive about the idea of a garment-integrated sensor system for early stress detection. In addition to detecting (triggers of) stress and decreasing challenging behavior, the sensor system was also deemed useful as a tool for evaluating intervention effects and the impact of health care professionals’ approach on residents, as well as an aid to increase self-regulation in residents. However, participants also expressed a few potential disadvantages or concerns. All participant groups, except people with mild ID, expressed concern that the sensor system could contribute to a reduction in human contact. In previous research, family caregivers perceived health care technologies as less desirable and were less willing to adopt technologies, due to this same fear of a decrease in social contact and human interaction [44,45], and health care professionals from the ID setting expressed this fear by emphasizing that a technology should not replace human contact [46]. Maintaining human contact in the provision of care is thus of great importance. Moreover, person-centered care and human contact in the provision of care has been proven beneficial for people with dementia and people with ID [47,48]. The use of technologies, including the use of a garment-integrated sensor system, should therefore be seen as complementary to human care to facilitate person-centered care, not as a substitute. It is important to communicate this clearly to all involved to help potential users make an informed decision and to stimulate successful adoption and implementation of the system [49]. Future studies should explore how the system might affect the caregiver-resident dynamic to provide insight into its social implications.

Participants in this study emphasized the importance of user friendliness, discretion, and comfort in wearing the sensor system. Customization was deemed essential, as each user would have unique needs and preferences. Both the garment and the user interface, including alarms and display features, should be customizable to accommodate individual requirements. Customization could be achieved by offering more than 1 garment option, different-colored garments, options to change the sound of the alarm (or no sound), and adjustable display features. Failure to tailor the system to the user’s needs and preferences was anticipated to increase the user’s stress levels and decrease acceptance. Previous research has shown that customization can enhance the user acceptance [50,51] and perceived value [52] of health care technologies. Increased acceptance and perceived value, in turn, can positively impact implementation success [31,52]. Moreover, customization aligns with the movement toward person-centered care for both people with dementia and people with ID in the Netherlands [53,54] and worldwide [55]. Future research should explore methods for tailoring interventions to accommodate the specific needs and abilities of individuals across a spectrum of dementia severity and ID. This may involve conducting additional assessments and consultations with stakeholders to better understand their unique requirements. Other suggestions for future research include examining strategies for ensuring the feasibility and effectiveness of personalized approaches in diverse care settings, and assessing the scalability and sustainability of personalized interventions, exploring how they can be replicated and adapted to serve larger populations, while maintaining their focus on customization. This involves developing flexible frameworks and protocols that can be easily adapted to different contexts.

Several factors were identified as important for implementing the garment-integrated sensor system in LTC settings, including perceived added value, costs, education, acceptance, privacy, and data security. These factors align closely with Greenhalgh’s technology implementation model [31]. Importantly, training and education materials may be developed with the ongoing involvement of the intended users to understand their needs and knowledge gaps. Short and clear written guidelines, instructions, and handouts appear to be important facilitators that could be easily implemented. Training appears to be an important factor for future implementation as it can address personal and psychological barriers, and without training, health care professionals tend to feel low self-efficacy when using any digital health technologies, resulting in negative attitudes toward these technologies [56].

Notably, our participants emphasized the significance of privacy and data security, which were not explicitly addressed in Greenhalgh’s model. However, this finding is consistent with a previous study where privacy concerns deterred family caregivers from adopting technology [57]. In our study, participants initially highlighted privacy as a critical requirement for using the sensor system, but after further discussion, they decided they valued the ability to measure stress more than maintaining strict privacy. They reasoned that privacy may be less of a concern in LTC, as residents often rely on assistance for daily tasks, which reduces their privacy. Additionally, participants did not perceive the health information generated by the sensor system, such as the heart rate, as an invasion of their privacy. This aligns with previous research that reported elderly individuals prioritized help over privacy concerns [58] and did not consider transmitted medical data from wearables as private [59]. Although participants acknowledged privacy as a potential barrier to implementation, they did not perceive the garment-integrated sensor system as privacy limiting. Nonetheless, it is crucial for the system to comply with privacy regulations, and data must always be properly secured.

Given the absence of consensus, determining the optimal data storage duration should be approached on a case-by-case basis. Nonetheless, leveraging longitudinal data could significantly enhance the development of individualized care plans and interventions. Specifically, these data could be used to identify patterns, triggers, and trends over time, informing personalized strategies to mitigate stress and improve overall well-being for people with dementia or ID. Hence, incorporating such longitudinal insights into care practices has the potential to enhance the effectiveness and responsiveness of interventions, ultimately leading to better outcomes for those under care. Future longitudinal studies should assess the long-term impact of using the garment-integrated sensor system for stress prediction in LTC residents and their caregivers. A comparison with existing stress detection technologies might highlight the proposed system's unique advantages.

### Strengths and Limitations

A strength of our study is the inclusion of all stakeholders, including people with dementia and people with ID, as well as family caregivers and health care professionals. This provided a diverse range of opinions and experiences, which enhanced the robustness of our findings. Moreover, conducting focus groups at different time points and sharing and discussing the results from previous focus groups with the participants further increased the internal validity of the findings. Lastly, a standard operating procedure for all focus groups allowed for consistency and increased reliability.

However, the inclusion of only 4 community-dwelling people with mild-to-moderate dementia, as well as a lack of participants with more severe dementia, could be seen as a limitation in this study. The latter is recognized as a common barrier in dementia assistive technology research [32]. Two recent reviews on patient and public Involvement (PPI) in dementia research provide useful strategies to facilitate PPI [60,61]. In addition, recruiting via the panel of Alzheimer Netherlands and conducting 3 rounds of focus groups may have resulted in selection bias, reaching the more motivated and less burdened participants. Moreover, most participants in this study had (several years of) experience with technology. Previous experience with technology may influence the perceptions of individuals [62], which limits generalizability. Despite its limitations, this study contributes to identifying the requirements for developing a garment-integrated sensor system for people with dementia and people with ID.

### Conclusion

Although a few potential disadvantages were mentioned, people with dementia, people with ID, health care professionals, and family caregivers were mostly positive about the concept of a garment-integrated sensor system for early stress detection in people with dementia and people with ID and expected it to be able to decrease stress and challenging behavior, for example, by identifying triggers and evaluating intervention effects. It is important that the garment-integrated sensor system meet several basic system requirements (washable and safe) and be user friendly and customizable to meet the specific preferences and needs of its users to increase user acceptance. Additionally, the sensor system should demonstrate a clear added value for health care professionals and residents, and education and protocols describing proper use of the system should be provided to all users to ensure successful implementation. Developing user-friendly training material for residents and caregivers could facilitate smoother adoption. The next crucial step involves (pilot-)testing the new wearable sensor system in LTC residents with dementia and residents with ID to evaluate its acceptability, comfort, and impact on stress and challenging behavior and to further validate the sensor system.

## Abbreviations

AWVB: Academic Collaborative Center Living with an intellectual disability
ID: intellectual disability
LTC: long-term care
UNC-ZH: University Network for the Care sector Zuid-Holland
